# Supplementary material for: Neurotensin Gene rs2234762 C>G Variant Associates with Reduced Circulating Pro-NT Levels and Predicts Lower Insulin Resistance in Overweight/Obese Children
Source: Int J Mol Sci. 2023 Mar 30;24(7):6460. doi: 10.3390/ijms24076460 (PMC10095103; doi:10.3390/ijms24076460)
Supplement: Supplementary file 1 [file ijms-24-06460-s001.zip › ijms-2283026-supplementary.pdf]

Table S1. Bivariate analyses between plasma pro-NT and clinical/biochemical parameters

	Correlation coefficient	p-value
**Age (years)**	0.195	0.019
**BMI (Kg/m2)**	0.075	0.367
**SBP (mm/Hg)**	0.035	0.678
**DBP (mm/Hg)**	0.087	0.311
**FBG 0′ (mg/dl)**	-0.058	0.487
**FBG 120′ (mg/dl)**	-0.037	0.659
**FBI 0′ (μUI/ml)**	0.001	0.990
**FBI 120′ (μUI/ml)**	0.131	0.115
**TC (mg/dl)**	-0.035	0.672
**HDL-C (mg/dl)**	-0.063	0.447
**LDL-C (mg/dl)**	-0.019	0.824
**TG (mg/dl)**	0.166	0.046
**AST (U/L)**	0.030	0.727
**ALT (U/L)**	0.053	0.533
**HOMA- β**	0.017	0.842
**HOMA-IR**	-0.006	0.943
**ISI**	-0.075	0.371
**SPISE**	-0.14	0.091

Abbreviations: BMI, body mass index; SBP, systolic blood pressure; DBP, diastolic blood pressure; FBG, fasting blood glucose; FSI, fasting serum insulin; TC, total cholesterol; TG, triglycerides; HDL-C, high-density lipoprotein; LDL-C, low-density lipoprotein; AST, alanine aminotransferase; ALT, aspartate aminotransferase; HOMA-IR, homeostatic model assessment of insulin resistance; HOMA- β, homeostatic model assessment for β-cell function; ISI, insulin-sensitivity index; SPISE, single-point insulin sensitivity estimator.
